# Personas sordas y lengua de señas: un enigma en odontología

**DOI:** 10.21142/2523-2754-1004-2022-135

**Published:** 2023-12-26

**Authors:** Mirian Elizabeth Muñoz Quispe, Michaell Jeremy Mendoza Cóndor, Jorge Enrique Cahuana Vílchez, Manuel Antonio Mattos-Vela

**Affiliations:** 1 Facultad de Odontología, Universidad Nacional Mayor de San Marcos. Lima, Perú. Mirian.munoz@unmsm.edu.pe, michaell.mendoza@unmsm.edu.pe, jorge.cahuana@unmsm.edu.pe, mmattosv@unmsm.edu.pe Lima Perú Mirian.munoz@unmsm.edu.pe michaell.mendoza@unmsm.edu.pe jorge.cahuana@unmsm.edu.pe mmattosv@unmsm.edu.pe

**Keywords:** sordera, barreras comunicativas, accesibilidad, atención dental, deafness, communication barriers, accessibility, dental care

## Abstract

La lengua de señas es el principal medio de comunicación de las personas sordas; consiste en la combinación de movimientos manuales, corporales y faciales con significados es.pecíficos. Entre las principales barreras a las que se enfrenta esta comunidad, resaltan la falta de comunicación y la escasez de servicios de salud, sumada a la inexperiencia y el desconocimiento de la lengua de señas por parte del profesional de la salud. El presente artículo tiene como objetivo dar a conocer la importancia y necesidad de la lengua de señas en la atención clínica odontológica y la formación profesional, así como de estrategias, herramientas y recomendaciones para una mejor atención de las personas sordas. Se incluyeron bases de datos como SciELO, Google Scholar, PubMed y ScienceDirect durante los años 2007 y 2021. Se concluyó que las principales barreras durante la comunicación son el poco dominio de lengua de señas por parte de los odontólogos, el poco acceso a intérpretes y las dificultades para la obtención de citas, lo que repercute en la susceptibilidad a desarrollar enfermedades bucodentales. La inclusión de cursos de lengua de señas dentro del currículo universitario aporta beneficios académicos y en la ética profesional; aun así, es necesario un seguimiento que corrobore el cumplimiento de los parámetros solicitados. Entre las principales herramientas y estrategias, el uso de videos educativos, el acompañamiento de intérpretes y -en vista del desarrollo tecnológico- el uso de aplicativos móviles pueden facilitar la comunicación. Asimismo, se plantea un conjunto de recomendaciones para el abordaje de pacientes sordos adultos y niños.

## Abstract (en)

Sign language is the main means of communication for deaf people; consists of the combination of manual, body and facial movements with specific meanings. Among the main barriers faced by this community are the lack of communication, shortage of health services, added to the inexperience and lack of knowledge of sign language by the health professional. This article aims to raise awareness of the importance and need of sign language in dental clinical care and professional training, as well as strategies, tools and recommendations for better care for deaf people. Databases such as SciELO, Google Scholar, PubMed and ScienceDirect were included during the years 2007 and 2021 It was concluded that the main barriers during communication are the little knowledge of sign language in dentists, the little access to interpreters and difficulties in obtaining appointments; affecting the susceptibility to develop oral diseases. The inclusion of sign language courses within the university curriculum provides academic and professional ethics benefits; however, follow-up is necessary to confirm compliance with the requested parameters. Among the main tools and strategies, the use of educational videos, the accompaniment of interpreters and, in view of technological development, the use of mobile applications, can facilitate communication. Likewise, a set of recommendations is proposed for the approach of adult and child deaf patients

## INTRODUCCIÓN

La lengua de señas (LS) es un mecanismo de comunicación no verbal llevado a cabo por la mayoría de los individuos sordos, con dificultades de audición o sin la capacidad de hablar ^1^. A lo largo del mundo, las LS suelen ser distinta para cada país; sin embargo, las personas sordas (PS) logran comprenderse debido a la representatividad de las señas utilizadas ^2^. 

La comunidad de PS puede presentar dificultades tales como falta de comunicación, interacción social, escasez de servicios en salud y -en la atención odontológica- el miedo, la falta de empatía y el poco abordaje en LS por parte del profesional. Diversos estudios señalan que el conocimiento en LS en el odontólogo ayuda a un mejor entendimiento para un adecuado diagnóstico y tratamiento, mientras que su desconocimiento es percibido por los pacientes como causa de discriminación e incomprensión ^3^^-^^5^.

Debido al poco acceso en educación acerca de la higiene oral y una escasez de profesionales aptos para su abordaje, las PS son más susceptibles a enfermedades bucodentales como la caries dental. En escuelas de Odontología y Farmacia se ha implementado el curso de LS como electivo u obligatorio, lo que ha dado resultados beneficiosos en la atención estomatológica, el desarrollo de empatía y el aumento del criterio ético ^5^^,^^6^.

Estudios realizados en América Latina y Asia señalan que los pacientes sordos han presentado un déficit en la atención estomatológica, tanto del personal que los atiende en la recepción como del mismo odontólogo ^4^^,^^7^. Es importante que el profesional tenga una buena relación con el paciente sordo, empezando por conocer las reglas básicas y aspectos generales para una mejor atención, lo que genera un ambiente cálido y la confianza para conocer más acerca de las dolencias que lo aquejan ^3^.

La atención odontológica en PS es escasa e inadecuada en la mayoría de los casos, debido al poco énfasis en la inclusión de comunidades minoritarias y la limitada enseñanza impartida, en este aspecto, a los profesionales de la salud durante su formación universitaria. Por tal razón, esta revisión bibliográfica tiene como objetivo dar a conocer la importancia y necesidad de la LS en la atención odontológica y en la formación profesional, además de contribuir con estrategias, herramientas y recomendaciones para una mejor atención de las PS.

## MATERIALES Y MÉTODOS

Se realizó una búsqueda bibliográfica en las bases de datos SciELO, Google Scholar, PubMed y ScienceDirect. Los descriptores utilizados fueron los siguientes: lengua de señas, sordos, pérdida de la audición, odontología, salud, atención, educación, protocolos, estrategias, las combinaciones entre ellas y sus equivalentes. La selección de artículos tomó en cuenta los siguientes criterios de inclusión: artículos en español, portugués e inglés; artículos con un intervalo de tiempo entre 2007 y 2021; artículos originales; revisiones sistemáticas; artículos especiales; artículos de reflexión; artículos destacados; artículos con abordaje en LS en aspectos como la educación, la atención al paciente, las recomendaciones, los protocolos, las herramientas de comunicación y el análisis del conocimiento entre los profesionales de la salud. Como criterios de exclusión estuvieron las cartas al editor, las editoriales, la literatura gris y los artículos que no cumplieran con el rango de tiempo establecido. Luego de la búsqueda, se seleccionaron 40 artículos, con base en su relevancia y cumplimiento de los objetivos de la revisión bibliográfica.

### Lengua de señas: descripción histórica

El lenguaje es un rasgo del sistema nervioso que depende de una vía de entrada que no se limita particularmente a señales sonoras, sino que incluye estímulos visuales ^8^. La LS es un mecanismo de comunicación no verbal empleado por la mayoría de los individuos sordos, con dificultades de audición o sin la capacidad de hablar. Consiste en la combinación de gestos, expresiones faciales y movimientos corporales con significados específicos, correspondientes a entradas visuales sensoriales ^1^.

Los registros históricos indican que la LS tiene un posible inicio en el siglo IV a. C., pero, como todas las lenguas, surgió como el resultado de interacciones poblacionales constantes ^8^^,^^9^. Para el siglo XVII, la filosofía y la religión seguían el concepto de que el oído, medio de transmisión del sonido, era la base del conocimiento; por lo que los individuos sordos y mudos se consideraban incompetentes, sin capacidad de aprendizaje ni “alcance de salvación”. Esto último cambia en el siglo XVIII, cuando Charles Michel estableció la primera escuela para sordos ^8^. No obstante, es a partir de la década de 1960 que las LS han ido tratándose como un lenguaje plenamente desarrollado ^9^.

Si bien existe una superposición de LS empleadas por diversas culturas, no se puede asegurar que es universal; por el contrario, se estiman más de 300 variaciones de LS a lo largo del mundo ^1^. De esta forma; las comunidades que comparten una misma lengua hablada no necesariamente poseen LS similares ^9^.

La LS se compone de un conjunto de gestos con una o dos manos correspondientes a palabras, letras o números: la mano dominante lleva a cabo los movimientos primarios, mientras que la no dominante, simétricos o auxiliares. El deletreo manual se emplea para nombres propios y palabras sin equivalentes. Asimismo, los movimientos faciales (ojos, boca, cejas) ayudan a transmitir el sentido de la oración ^1^.

Actualmente, alrededor de 70 millones de PS en todo el mundo consideran la LS como “su primera lengua” o “lengua materna” ^9^.

### ¿Sordera o pérdida de la audición?

Sordera y pérdida de la audición, en resumen, son términos que se incluyen uno al otro; pese a ello, es común observar que existe una confusión entre ambos, al tomarse como sinónimos.

Según la OMS, la pérdida de audición implica que una persona no es capaz de oír tan bien como aquella que posee el sentido del oído normal (umbral igual o mejor que 20 dB). Esta puede ser leve (26-40 dB), moderada (41-60 dB), grave (61-80 dB) o profunda (> 80 dB); afectar a uno o ambos oídos y generar dificultades en la comunicación verbal ^10^^,^^11^. El término “sordera” supone un padecimiento de pérdida de la audición profunda, es decir, no es posible oír o, por el contrario, solo hacerlo de forma mínima, por lo que usualmente se comunican a través de LS ^10^. Cabe remarcar que no existen recomendaciones establecidas para evitar el uso de “sordo” para aquellos que consideran el término despectivo o discriminatorio, por lo que ello se limita a la preferencia personal del profesional ^11^.

### Salud oral en PS

El poco acceso a los servicios dentales, la carencia de educación en el cuidado de la salud oral y el tratamiento dental aumenta la susceptibilidad de las PS ante patologías bucodentales, como la enfermedad periodontal y la caries dental ^12^^-^^17^ (Tabla 1). Se evidenció que, a pesar de usar cepillo y pasta dental, las PS no realizaban una adecuada técnica de cepillado; asimismo, conforme aumentaba la edad, perdían un mayor número de piezas dentales a causa de la caries ^12^^,^^16^. Por tanto, es necesario promover medidas preventivas y estrategias que favorezcan la disminución de los factores de riesgo (higiene oral y dieta), así como brindar programas que incentiven a mejorar y reforzar la salud bucodental en las PS.

**Tabla 1:** Prevalencia de enfermedad periodontal y caries dental en PS

	Autor	Año	País	Caries dental	Enfermedad periodontal	Otros
Niños	Jnaneswar *et al*.^12^	2017	India	104 (19,3%)	129 (23,9%) SAS*	Candidiasis (18%) Lengua saburral (8%) Queilitis angular (2%) Macroglosia (6%)
					255 (47,2%) CD**	
	Yazeed Al-Qahtani *et al*.^13^	2017	Arabia Saudita	61 (97%)		
	García Pérez *et al*.^14^	2018	México			
	Suma *et al*.^15^	2011	India	32 (42%)	24 (32%) Gingivitis	
Adultos	Alkahtani *et al*.^16^	2019	Arabia Saudita	120 (82,2%)	86 (60,1%) Inflamación gingival moderada	
	Qaanita^17^	2019	Sudáfrica		26,09% Periodontitis	

* SAS: sangrado al sondaje. ** CD: cálculo dental

Por otro lado, el rol de los cuidadores en los niños sordos es fundamental para la sostenibilidad de un buen estado de salud oral, ya que, a pesar de que las PS tengan pocos conocimientos acerca de higiene oral, han logrado mantener niveles aceptables o excelentes debido a la atención de los padres ^14^. 

### Barreras en la atención odontológica de las PS

El éxito de la atención odontológica depende, principalmente, de una buena comunicación y relación paciente-profesional; sin embargo, cuando se trata con una PS todo ello se ve comprometido. El desconocimiento de los profesionales acerca de las LS, sumado a la falta de experiencia y el miedo a tratar con pacientes de este tipo puede generar desconfianza por parte de estos, que lo perciben como un acto discriminatorio ^3^^,^^18^^,^^19^. Un estudio realizado en Venezuela indicó que 26 de las 27 PS participantes aseguraron haberse sentido discriminadas, y una de las causas fue el desconocimiento de la LS ^3^. 

Algunos autores han observado que las PS tienen la capacidad de comunicarse mediante la lectura de los labios, como recurso adicional para una mejor comprensión de su diagnóstico y tratamiento, por lo que el profesional debe tomar en consideración estos aspectos ^20^^,^^21^. Cabe decir que no todos las PS poseen esta habilidad e inclusive aquellos que saben hacerlo prefieren no optar por esta estrategia ^21^. 

Pese a que en algunos países se ha implementado legalmente el servicio de un intérprete como intermediarios comunicacionales, se ha reportado que muchas clínicas rechazan citas cuando se trata de un paciente sordo. Se comparó el acceso a citas médicas y dentales entre pacientes sordos simulados y pacientes que podían oír, y se obtuvo como resultado que los pacientes simulados que podían oír presentaban casi dos veces más probabilidades de asegurar citas, a diferencia de los pacientes sordos simulados ^7^. Ello genera desmotivación, frustración, impotencia y desconfianza, ya que, al ser rechazados constantemente, solo buscan ayuda en caso de enfermedad y no de prevención; por ello, el odontólogo debe plantearse nuevas estrategias tales como escritura, dibujos o el uso de aplicativos que mejoren la atención clínica ^19^.

Ciertos estudios han advertido que involucrar al intérprete como una tercera persona en la comunicación, ya sea un familiar o personal del Estado, puede generar desconfianza o falta de confidencialidad. por lo que las PS pueden ocultar información relevante para el diagnóstico y tratamiento ^20^^,^^22^. En contraste, en algunos casos, los pacientes se han sentido más seguros con la presencia de un familiar que cumple la labor de intérprete ^19^^,^^22^.

### Formación odontológica con lengua de señas. Importancia de LS en la formación odontológica

Numerosos autores sugieren la necesidad de incluir cursos de capacitación en LS como parte de la formación universitaria del profesional de la salud, para satisfacer la atención en pacientes sordos ^3^^-^^6^; no obstante, se ha reportado que, si bien se han añadido cursos de LS, no se han realizado de manera adecuada. Asimismo, es importante considerar que un alto porcentaje de odontólogos o estudiantes de odontología no conocen ni están capacitados en LS.

Santos *et al*. ^4^ hallaron que más de la mitad de los estomatólogos evaluados no conocían las reglas de comunicación ni los aspectos de atención a las PS, así como los estímulos negativos y los motivos de insatisfacción de esta comunidad. Al Jaafar *et al*. ^23^ encontraron diferencias no significativas entre los diferentes niveles de los estudiantes con respecto al manejo de la LS. De esta forma, la situación de las PS ante problemas odontológicos presenta un panorama cada vez más incierto, con profesionales sin las competencias necesarias para establecer un diagnóstico y tratamiento adecuado, el cual depende muchas veces de una buena interacción paciente-profesional, en la que la comunicación resulta un punto esencial.

Las universidades brasileñas tienen como reglamento añadir el curso de LS en las carreras de ciencias de la salud, pese a ello, Souza *et al*. ^24^, al analizar los planes de estudios de 35 de las escuelas de farmacia de las instituciones federales de educación superior, hallaron que solo 18 ofrecían el curso de LS de manera electiva y 14 de ellos se daban de manera 100% teórica. Además, al evaluar el contenido de los cursos, solo 12 consideraron los aspectos clínicos, educativo y sociales; y tan solo cuatro, los aspectos legislativos y las políticas públicas. Esto expone la ausencia de contenidos y la falta de garantías para que los estudiantes adquieran las competencias necesarias para tratar con las PS. 

Ribalta *et al*. ^25^ diseñaron un curso virtual de LS para estudiantes de medicina con el fin de auxiliar la comunicación de pacientes o familiares que requieran ser atendidos. Los comentarios que obtuvo la investigación fueron beneficiosos, y se recalca la viabilidad de su aplicación para favorecer la comunicación con las PS, forjando valores y actitudes relativas al humanismo y la solidaridad. Por lo tanto, la implementación de cursos de LS mediante plataformas virtuales, como complemento de aprendizaje en la formación de los estudiantes de odontología, puede ser favorable para mejorar su formación.

Un estudio realizado por Santos y Novoa ^26^, en estudiantes de odontología de La Habana, reconoció que el plan de estudios no aborda con suficiente detalle la atención de los pacientes sordos, por lo que considera importante integrar un curso de LS con el fin de disminuir la brecha de comunicación en la atención odontológica. Ante esta situación, la implementación de cursos obligatorios o electivos de la LS para estudiantes de pregrado en odontología puede ser significativamente beneficioso en su formación, ya sea en el ámbito de las competencias clínicas de la atención odontólogo-paciente, como en forjar la empatía y el criterio ético ante PS ^5^^,^^26^. 

### Estrategias y herramientas para el manejo de PS

El trabajo del profesional con las PS necesita, muchas veces, de herramientas para lograr una atención exitosa. Por ende, es importante que las instrucciones impartidas se transmitan de manera que puedan conjugar sus requerimientos. Múltiples estudios resaltan las desventajas que posee la comunidad de PS con respecto a su estado de salud bucal; en consecuencia, es importante considerar la higiene oral como un factor esencial para la prevención de enfermedades bucodentales a futuro ^12^^-^^17^. 

La LS representa el método de comunicación más eficaz para el manejo de PS, seguido por el uso de videos educativos y el acompañamiento de intérpretes, que pueden ser de ayuda para establecer satisfactoriamente algunas indicaciones de salud oral ^20^^,^^27^. 

Fageeh *et al.*^28^ observaron diferencias significativas entre el conocimiento de salud bucal de personas con discapacidad auditiva en Arabia Saudita, divididas en dos grupos: 1) posterior a la aplicación de videos educativos, y 2) posterior al uso de folletos impresos, acerca de instrucciones de higiene oral en LS. La aplicación de videos educativos demostró ser más eficaz comparada con el empleo de folletos, con relación al conocimiento de salud bucal en la población.

Sudhindra *et al*. ^29^ compararon el impacto de instrucciones de higiene oral a través de LS y un video educativo validado para niños sordos. El video tuvo la participación de PS explicando los efectos de una inadecuada higiene oral tanto en la salud como en sus relaciones sociales, con el objetivo de servir como modelo para los participantes. Se hallaron diferencias no significativas entre los índices de placa y el estado gingival de la muestra posintervención; por ende, para ambas técnicas, la efectividad fue similar.

Es recomendable considerar herramientas lúdicas de enseñanza para brindar instrucciones sobre salud oral a niños sordos, con el fin de generar una concientización que pueda prevalecer en sus hábitos y, por consiguiente, en etapas futuras de su vida ^30^. Ávila-Curiel *et al*. ^31^ realizaron una intervención mediante un juego de cartas denominado Loti-denti, para aumentar los conocimientos sobre higiene oral y disminuir el índice IHO-S en un grupo de niños sordos, y lograron un incremento en los valores de IHO-S en el nivel bueno (12% al inicio y 64% tras la intervención). 

El constante desarrollo de las aplicaciones móviles ha generado nuevas oportunidades para el acceso a la salud, forjando cada vez más lo que representará el futuro de la atención médica, con un entorno más inclusivo para las PS ^32^. Pérez-Baquero *et al*. ^33^ desarrollaron un prototipo de aplicativo móvil denominado Dentiseñas-Colombia, con el fin de facilitar la comunicación entre el odontólogo y las personas con discapacidad auditiva durante la primera consulta, el consentimiento informado y la fase de prevención y promoción en salud oral. Se adaptaron frases y posibles respuestas en LS, grabándose videos representativos que ayuden a la comprensión del profesional en odontología. 

### Recomendaciones en el abordaje de PS

La atención odontológica de las PS ha cobrado mayor protagonismo con el pasar de los años, debido a que los profesionales no se encuentran capacitados para atender a personas con problemas de comunicación ^34^. Por ende, se debería dar a conocer algunos conceptos generales que el odontólogo deberá tener en cuenta cuando se presente un PS. Las recomendaciones para mejorar la atención de los pacientes sordos se muestran en las Tablas 2 y 3.

**Tabla 2:** Recomendaciones para el manejo de pacientes sordos adultos

Recomendaciones: pacientes sordos adultos
- La presencia de una deficiencia auditiva y habilidades que puedan mejorar la comunicación deben estar relatadas en la historia clínica. - Mirar de frente al paciente, para generar confianza entre este y el profesional. - Explicar, mediante señas básicas, todo lo que está sucediendo en el tratamiento; tener en cuenta que una PS tiene la vista y el tacto muy desarrollados. - Mostrar los instrumentos que se van a utilizar en el tratamiento. • Aclarar que las vibraciones que realizan estos instrumentos, como las piezas de altas y bajas velocidades, son normales. • Reducir la velocidad de las piezas de alta o baja y colocarlas en su mano para que pueda conceptualizar la vibración y no sienta temor cuando se encuentre en su boca. - En caso se requiera, acudir con un intérprete (es preferible que sea un familiar del paciente). Otras alternativas pueden ser el uso de imágenes referenciales y videos. - El paciente puede realizar gestos o señas con las manos cuando se sienta incómodo durante el tratamiento (dolor, acumulación de agua en la boca). - Evitar estímulos desfavorables (dolor, cansancio, etc.) puede mejorar la percepción de la experiencia del paciente. - Realizar sesiones cortas y con horarios flexibles.

**Tabla 3:** Recomendaciones para el manejo de pacientes sordos pediátricos

Recomendaciones: pacientes sordos pediátricos
- La comunicación toma importancia al realizar la anamnesis, ya que es necesario conocer el grado de discapacidad e interacción que tiene el paciente. Por ejemplo, debe anotarse el grado de severidad de comunicación y medir el comportamiento según la escala de Frankl ^35^. - El abordaje conductual del odontólogo hacia su paciente debe ser asistido mediante técnicas. Por ejemplo, la técnica de mostrar, oler, tocar y hacer. - El odontólogo deberá reconocer los grados de ansiedad de sus pacientes debido a la falta de comunicación, ya que de esto dependerá el éxito del tratamiento. Ejemplo: pacientes inseguros, miedosos, retraídos, temerosos o no colaborativos. - Programar citas cortas y a disposición del paciente, ya que son muy poco tolerantes a la espera. - Se debe mostrar al niño todos los instrumentos que se van a utilizar, así como qué función realizan. Ejemplo: los efectos de los instrumentos vibratorios son normales. Asimismo, se recomienda bajar la intensidad de la pieza de baja y alta, y colocarla en su mano para que se familiarice con la vibración y no sienta desconfianza. - El odontólogo y el asistente dental deben ganarse la confianza del niño mediante señas, juegos lúdicos, expresiones faciales. - Se recomienda, para tratamientos complejos, estar bien capacitados y realizar la interconsulta con el odontopediatra. - El uso de la técnica de “modelado”, que puede ser realizada por un pariente, ya sea la madre o un hermano, genera una mayor seguridad en ellos ^36^. - Apoyarse con material audiovisual para explicar el tratamiento, con el fin de no generar inseguridad. Ejemplo: videos, cartas, collage, dibujos. - Antes de iniciar la rehabilitación oral, se recomienda acordar con la familia adaptar señas durante la rehabilitación. Ejemplo: levantar el dedo significa que el niño no siente inseguridad, cerrar la boca con aire significa que quiere botar el acúmulo de agua en la boca ^37^.

## CONCLUSIÓN

Con base en la minuciosa revisión de la literatura, se puede afirmar que los principales obstáculos en la comunicación odontólogo-persona sorda es el desconocimiento de la lengua de señas; debido a ello, esta última se encuentra más susceptible a enfermedades bucodentales como caries dental y enfermedad periodontal. La revisión nos demuestra que, en el campo educativo, la implementación del curso de lengua de señas ha resultado favorable, debido al aumento de la empatía, el criterio ético y la confianza al tratar con una PS; sin embargo, se debe realizar un seguimiento para verificar el cumplimiento del contenido. Si bien es cierto que la principal herramienta es la LS, se recomienda usar recursos como videos educativos y juegos de cartas que ayuden a fortalecer los conocimientos de higiene oral. Asimismo, la intervención de un intérprete vulnera la privacidad de la persona sorda y, a veces, puede ser perjudicial en el diagnóstico y, por ende, en el tratamiento; no obstante, a otros pacientes les resulta confortable, ya que de esta manera pueden expresarse. Se recomienda la enseñanza de manera electiva u obligatoria de la LS en las facultades de odontología y la instauración de programas preventivos.

## Financiamiento:

Autofinanciado
